# Removal of gaseous toluene using immobilized *Candida tropicalis* in a fluidized bed bioreactor

**DOI:** 10.1007/s13205-011-0015-7

**Published:** 2011-07-14

**Authors:** Zubair Ahmed, JiHyeon Song

**Affiliations:** 1 Department of Civil Engineering, Faculty of Engineering, King Abdulaziz University, P.O. Box 80204, Jeddah, 21589 Kingdom of Saudi Arabia; 2 Department of Civil and Environmental Engineering, Sejong University, Kwangjin-gu, Seoul, 143-747 Republic of Korea

**Keywords:** *Candida tropicalis*, Gaseous toluene, Immobilized yeast, Fluidized bioreactor, Elimination capacity

## Abstract

A pure yeast strain *Candida tropicalis* was immobilized on the matrix of powdered activated carbon, sodium alginate, and polyethylene glycol (PSP beads). The immobilized beads were used as fluidized material in a bioreactor to remove toluene from gaseous stream. Applied toluene loadings were 15.4 and 29.8 g/m^3^ h in Step 1 and Step 2, respectively, and toluene removal was found above 95% during the entire operation. A continuous pH decline was observed and pH of the suspension was just above 6 in Step 2 but no adverse effects on treatment efficiency were observed. The CO_2_ yield values were found to be 0.57 and 0.62 g- in Step 1 and Step 2, respectively. These values indicate that a major portion of toluene-carbon was channeled to yeast respiration even at higher toluene loading. In conclusion, immobilized *C. tropicalis* can be used as a fluidized material for enhanced degradation of gaseous toluene.

## Introduction

Toluene (C_7_H_8_) is an aromatic hydrocarbon belonging to the BTEX group of hazardous volatile organic compounds (VOC), which includes benzene, ethylbenzene and xylene. The use of this, relatively water-soluble, aromatic hydrocarbon as a solvent in the production of paints, thinners, adhesives, inks and many pharmaceutical products, as an additive in gasoline mixtures to increase octane ratings, in benzene production, results in its subsistence in air and wastewater emissions associated with these industries.

Biofilters has been used for the removal of toluene in waste air. These biofilters used compost as media (Delhomenie et al. 2002; Lu et al. 2002; Vergara-Fernandez et al. 2007), packing with peat (Yoon and Park 2002), and agro-waste (Singh et al. 2006). Biofiltration of toluene has been well studied (Hwang et al. 2003; Peishi et al. 2004). In efficient biofilters organic pollutants are essentially converted into mineral end products while biomass is produced at low rates. Nevertheless, in real cases, excess biofilm develops within the porous medium, and thus causes clogging of the fixed-bed bioreactors (Delhomenie et al. 2002; Iliuta and Larachi 2004; Xi et al. 2006). Different methods of clogging control have been tested (Iliuta and Larachi 2004; Mendoza et al. 2004). However, applied methods such as air sparging, backwashing and filling/draining methods to prolong the operation period of such biofilters remained inefficient (Shim et al. 2002; Okamoto et al. 2003).

Suspended-growth reactors (SGRs) constitute an effective alternative to overcome the above mentioned limitations of biofiltration processes, being more reliable in terms of construction and process control ( Koutinas 2005). They can be advantageous in processes operated at high pollutant loading rates, where by-product accumulation is likely to occur, or during the degradation of chlorinated VOCs, where medium acidification severely reduces process performance (Lee et al. 2000; Munoz et al. 2007). Neal and Loehr (2000) demonstrated SGRs can achieve elimination capacities higher than biofilters for the treatment of toluene. SGRs represent nowadays a cost-effective alternative to bioscrubbers or biofilm-based bioreactors in processes requiring a rapid and reliable control of pH, nutrient addition or toxic metabolites accumulation (Bordel et al. 2008; Burgess et al. 2001).

Immobilized cell systems are considered better than the conventional waste treatment system for biodegradation of toxic VOCs because high densities of specialized microorganisms can be used in such systems. Immobilization technique has been used in biofilter for treatment of waste gases particularly for hydrogen sulfide and ammonia (Chung et al. 1996, 1997; Huang et al. 1996). Shim and Yang (1999) developed a fibrous-bed bioreactor containing the co-culture of *Pseudomonas putida* and *Pseudomonas fluorescens* immobilized in a fibrous matrix to degrade benzene, toluene, ethylbenzene, and *o*-xylene in synthetic waste streams. They found that for immobilized cells, the degradation rate was much higher than that of free cells and immobilized cells in the bioreactor that tolerated higher concentrations (>1,000 mg l^−1^) of benzene and toluene, and gave at least 16-fold higher degradation rates for benzene, ethylbenzene, and *o*-xylene, and a ninefold higher degradation rate for toluene. Nakao et al. (2000) used a packed column bioreactor containing the immobilized activated sludge gel beads to remove toluene, benzene or ethylbenzene as a model of the toxic volatile organic compounds (VOC) in gas phase. The packed column was efficient for 1 year to remove the VOC. Jianping et al. (2005) operated a gas–liquid–solid three-phase flow airlift loop bioreactor to treat air streams containing a mixture of ethyl acetate and ethanol. The activated sludge was replaced by biological membrane in the experiment. The elimination capacities in the airlift bioreactor were higher than those in the conventional compost-bases biofilters.

For biodegradation of toluene, *P*. *putida* has been widely used (Shim and Yang 1999; Jung and Park 2004). However, temperature and oxygen limitations could be a major hurdle in wider application of *P.putida* (Maestre et al. 2007). Furthermore, biological treatment processes used to remove and degrade volatile organic compounds (VOCs) from contaminated gases emitted by industrial operations or waste treatment processes are almost always subjected to transient loading conditions because of the inherently unsteady-state nature of the contaminant generating processes. The transient loading also reduces biomass activity in the bioreactor. Recently *Candida tropicalis* has been used for the biodegradation of VOCs by many researchers. Yan et al. (2005) isolated strain *C*. *tropicalis* from acclimated activated sludge and studied phenol biodegradation using a pure culture of *C. tropicalis*.

Although, combination of fluidized bed type reactor (FBR) and immobilized cell have been used for the treatment of VOC in contaminated water, the potential of this combination has yet to be explored for contaminated gas treatment, particularly for toluene in gas phase. In a previous study, we have demonstrated enhanced toluene removal by *C. tropicalis* due to adsorption of toluene on granular activated carbon (Ahmed et al. 2010). In this study, a pure strain of *C. tropicalis* was immobilized on a matrix of powdered activated carbon/sodium alginate/polyethylene glycol (PSP) mixture and used for biodegradation of toluene in fluidized bed type bioreactor. The toluene biodegradation was evaluated by monitoring the toluene removal efficiency at different toluene loading rates. In addition, carbon dioxide evolution, pH in suspension, and protein content in suspension as well as within the beads were also measured. Furthermore, short-term elimination capacities were also determined to evaluate the bioreactor performance.

## Materials and methods

### Culture of *C*. *tropicalis*

Strains of yeast *C. tropicalis* were obtained from two different sources; Korean Culture Center of Microorganisms (KCCM) and Korea Biological Resource Center (BRC). The strains were incubated on YM agar plates for 3 weeks before transferring to fresh media. After several cycles of regeneration, a small portion of each strain was transferred into a 120-mL serum bottle, containing 50 mL of nutrient solution. The nutrient solution consisted of a hydrocarbon minimal medium (HCMM) containing 1.36 g/L KH_2_PO_4_, 1.42 g/L Na_2_HPO_4_, 0.5 g/L (NH_4_)_2_SO_4_, 3.03 g/L KNO_3_, and a trace metal solution containing 0.25 mg/L FeSO_4_·7H_2_O, 0.18 mg/L MnCl_2_·4H_2_O, 0.02 mg/L CuCl_2_·2H_2_O, 0.04 mg/L ZnSO_4_·7H_2_O, 0.04 mg/L CoCl_2_·6H_2_O, 0.02 mg/L NiCl_2_·6H_2_O, 0.02 mg/L Na_2_MoO_4_·2H_2_O, and 0.02 mg/L H_3_BO_4_. Sterilized air was sparged into the suspension for 2 min, and then the bottle was tightly caped with a Teflon-coated septum. Then a 10 microliter of toluene was injected into the headspace of the serum bottle and incubated in a shaker at 20 °C. The concentration of toluene in the headspace was monitored until toluene was completely removed. The response of *C. tropicalis* from both origins was similar; therefore, the strain obtained from KCCM was used in the further experiments.

### Immobilization of yeast

In order to immobilize *C*. *tropicalis* on beads of powdered activated carbon (PAC)/sodium alginate/polyethylene glycol (PSP), 1 g of sodium alginate was dissolved in 20 mL of water at 60 °C and then 18 g of polyethylene glycol was added and dissolved. Then 30 μL of TEMED was added and pH was adjusted using 10% of acetic acid in the range of 6–8. After adjusting pH, 1 g of PAC was added and volume of the mixture was made up to 50 mL. A volume of 50 ml of concentrated *C*. *tropicalis* culture was added in the mixture and stirred for about 30 s. The mixture was immediately filled in the plastic tube (diameter 5 mm), dried first at room temperature for 10 min and then 30 min at 30 °C, and extruded with 50 mL syringe in the nutrient solution. Finally the beads were made by cutting it into 2–3 cm long pieces.

### Bioreactor systems and startup

In this study, a fluidized bioreactor was used. The bioreactor consisted of acrylic pipe with an internal diameter of 8 cm and effective height of 40 cm, corresponding to the working volume of 1.3 L. The liquid samples were drawn from the sampling ports located along the column. Compressed air was passed through a filtration device to remove moisture, oil and particulate matters. After purification, the compressed air was mixed with research grade toluene which was injected using a syringe pump. The air stream and injected toluene were allowed to mix in a mixing chamber in order to achieve uniform concentration at the inlet of the bioreactor. The bioreactors were operated continuously by feeding the toluene-loaded gas stream through a diffuser placed at the bottom of the column. The bubble rose through the column filled with the yeast culture and the nutrient/buffer solution.

### Bioreactor operation

The bioreactor was filled with 55 g of PSP beads and nutrient solution before start up. The fluidized bioreactor was operated in three different sets of operating conditions over a 21-day period, as summarized in Table 1. Prior to start-up, 55 g of PSP beads as fluidized material (4.2%, w/v) were added to the yeast/nutrient solution in the culture. During the first phase of bioreactor operation, the inlet toluene concentration was sequentially increased: 68 ± 6 and 132 ± 4 ppm_v_ in Steps 1 and 2, respectively, at a constant EBGRT of 1 min. About one-tenth of the liquid medium was replaced with fresh nutrient solution on a daily basis, while retaining the PSP beads within the bioreactor.

**Table 1:** Experimental conditions used in this study

Experimental phases	Description	Duration (days)	Toluene	
			Concentration (ppm_v_)	Loading (g/m^3^ h)
Step 1	Low toluene loading	8	68 ± 6	15.4
Step 2	High toluene loading	8	132 ± 4	29.8

## Analytical methods

To determine overall toluene removal efficiencies, duplicate gas samples were collected from the inlet and outlet sampling ports using 0.5-mL gas-tight syringes (Hamilton #1750, USA) with Mininert syringe valves (Supelco, USA). The grab samples were immediately analyzed using a gas chromatograph (GC) (Series 6890, Agilent, USA) equipped with a flame ionization detector (FID) and a capillary column (HP-5, Agilent, USA). The C/FID response was calibrated with six known toluene standards, and blank and one-point toluene standards were injected into the GC/FID prior to daily analysis for the purpose of quality assurance. The CO_2_ evolved from the bioreactor was directly measured using an infrared CO_2_ analyzer (LI-820, LI-COR, USA).

The liquid medium withdrawn from the column on a daily basis was analyzed for chemical oxygen demand (COD), optical density (OD), and protein content. An appropriate amount of homogenized sample was added to a COD vial (Hach, USA), and the COD was measured according to the closed reflux, calorimetric method in standard methods. To observe the OD, a UV-VIS spectrophotometer (Shimadzu, Japan) was used at a wavelength of 600 nm. The protein content of the liquid medium was measured using a protein micro assay kit (Bio-Rad, USA). Bovine serum albumin was used as the protein standard (Bio-Rad, product #500-0002). Prior to the protein measurement, yeast cells were disrupted and microbial protein was extracted by ultrasonication with an ultrasonic processor (Sonic VibraCell, Sonic and Materials Inc., USA) at an applied acoustic power of 30 W for 6 min. The cell suspension was kept in a salt–ice bath during cell disruption to prevent over-heating. All analysis was conducted in triplicate to ensure reproducibility of results. Standard deviations of the measurements were <10%.

## Results and discussion

In order to enhance biodegradation of toluene and to improve the bioreactor performance, immobilized *C. tropicalis* PSP beads were used as fluidized material. Powdered activated carbon (PAC), alginate, and polyethylene glycol are considered as good adsorptive materials; therefore, it was expected that adsorption of gaseous toluene onto the beads will provide sufficient time to yeast cells for enhanced biodegradation of toluene. The bioreactor was initially started with 55 g of PSP beads, with stepwise increase in the loading of toluene.

**Fig. 1:**
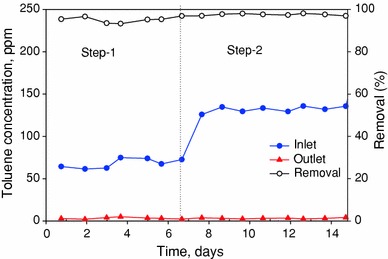
Removal of toluene in fluidized bed bioreactor

The biodegradation of toluene was almost complete when immobilized yeast cells were used in the bioreactor, as shown in Fig. 1. The removal efficiency was above 93% throughout the Step 1 and no significant acclimation period was observed. In Steps 2 and 3, the average removal efficiencies were 98 and 96%, respectively, with immediate establishment of pseudo steady-states conditions just after increasing the toluene loading.

**Fig. 2:**
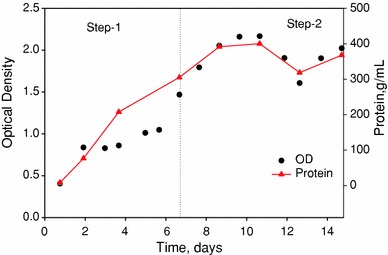
Biomass concentrations in terms of optical density and protein content in suspension of the fluidized bioreactor

**Fig. 3:**
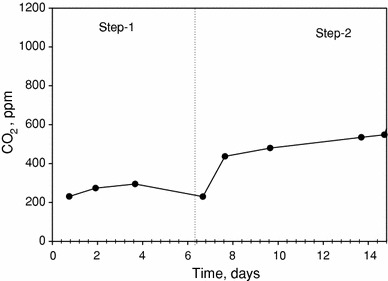
Evolution of CO_2_ from the fluidized bioreactor

**Fig. 4:**
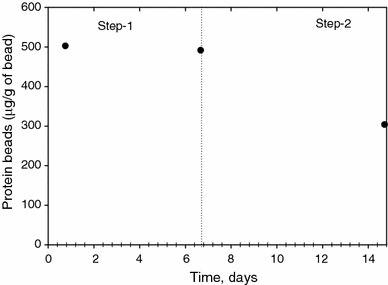
Protein content with the PSP beads

A sharp increase in the protein content in suspension of the bioreactor was also observed during all three steps (Fig. 2) which indicates that activity of *C. tropicalis* was also increased in suspension (Fig. 2). This increase of protein content was also verified by the increase of OD values of the suspension (Fig. 3). It can be suggested that the *C. tropicalis* population in the suspension increased because the mass transfer coefficient in the bioreactor was increased in the presence of PSP beads as well as due to slow release of adsorbed toluene onto the PSP, which then become available as a substrate for the yeast culture. The increased concentration of CO_2_ evolved from the bioreactor further supports our observation that the *C. tropicalis* activity increased by the addition of yeast immobilized PSP beads (Fig. 4). During Step 2, both OD and protein content in the suspension were decreased (day 10 to day 13) but reduction in toluene removal efficiency was not observed.

Furthermore, protein content within PSP beads was also monitored at the beginning of each step. In order to measure protein content within the beads, a bead was selected (in triplicate), grounded and then resuspended in 1 mL of distilled water. Then, microbial protein was extracted by ultrasonication with an ultrasonic processor and protein contents of the suspension were measured as described earlier. The protein content within the beads remains almost 500 μg/g after 8 days of operation, although, the protein content decreased to about 300 μg/g after 15 days of operation. The decrease of protein content was probably due to tear of beads and prolong operation. The beads were intact and in shape but the yeast cells released from the bead matrix were due to the vigorous shaking during fluidizing process.

**Fig. 5:**
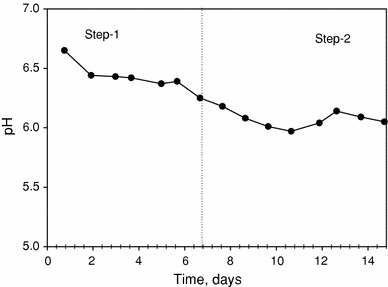
Changes in pH values in the fluidized bioreactor

The pH of the bioreactor was monitored throughout the operational period (Fig. 5). The initial pH of the medium was 6.8 which gradually declined with increased toluene loading. At the end of Step 1, the pH value was 6.18 and declined to 5.97 in the middle of Step 2 (day 11). After day 11, composition of buffer solution was slightly changed by substituting ammonium sulphate with ammonium phosphate. The pH value improved slightly but continues to decline. However, due to the change in buffer composition, a recovery of protein content was observed in the suspension (Fig. 4). The pH values were 6.05 at the end of Step 2. Despite the decline in pH values at higher toluene loading, the bioreactor performance was not affected and toluene removal efficiency was maintained above 95%. These findings indicate that the bioreactor using the immobilized yeast culture can maintain its toluene-degrading activity above pH 5; therefore, maintenance of neutral pH conditions in the bioreactor will be sufficient for stable operation of the fluidized bioreactor.

### Carbon balance

To evaluate toluene-carbon utilization by *C*. *tropicalis*, a CO_2_ yield was calculated. The CO_2_ yield values were found to be 1.90 and 2.07 g-CO_2_/g-toluene in Step 1 and 2, respectively, corresponding to 0.57 and , respectively, on a carbon-mass basis. It can be seen that the values increased with increase in toluene loading, indicating that more <43% of the toluene was utilized by the yeast cell through microbial respiration as indicated by the CO_2_ production in Step 1. Major portion of carbon was utilized by the yeast cells and less for synthesis due to immobilization of the yeast cells in the PSP matrix. In a previous study, the carbon utilization by the yeast cells was found to be  in a suspension growth bioreactor (Ahmed et al. 2010). It can be concluded that immobilization of the yeast cells enhances the biodegradation of toluene in the bioreactor used in this study.

## Conclusion

A pure yeast strain “*C*. *tropicalis*” was immobilized on matrix of powdered activated carbon, sodium alginate, and polyethylene glycol (PSP beads). The immobilized beads were then used as fluidized material in a bioreactor to remove toluene from gaseous stream. The biodegradation of toluene was enhanced due to the adsorptive nature of matrix on which the yeast cells were immobilized. During operation of the bioreactor, toluene loadings were 15.4 and 29.8 g/m^3^ h. Toluene removal was above 95% during the entire operation of the bioreactor. The bioreactor was operated for 15 days and protein content within the beads was slightly decreased, indicating that the yeast cell was viable with the beads during entire operation of the bioreactor. A continuous pH decline was observed and final pH of the suspension after 15 days of operation was just above 6, however, no adverse effects on the treatment efficiency were observed. The bioreactor can be operated at neutral pH range with optimum toluene removal performance. A carbon balance of toluene removal in the system indicates that relatively less portion of toluene-carbon was used for yeast cell synthesis and major portion of the carbon was utilized by the cell for respiration due to immobilization of yeast cells. Hence, immobilized *C. tropicalis* can effectively be used in a fluidized bioreactor for the removal of toluene from gaseous stream.
